# Use of technologies in thyroid surgery: Latin American Thyroid Society Surgical Affairs Committee Expert Opinion. Part 1

**DOI:** 10.20945/2359-4292-2024-0111

**Published:** 2025-03-27

**Authors:** Alvaro Sanabria, Jose Luis Novelli, Erivelto Volpi, Ana Voogd, Santiago Zund, Luiz Paulo Kowalski, Juan Pablo Dueñas

**Affiliations:** 1 Departamento de Cirugía, Facultad de Medicina, Universidad de Antioquia, Medellín, Colombia; 2 CEXCA, Centro de Excelencia en Enfermedades de Cabeza y Cuello, Medellín, Colombia; 3 Departamento de Cirurgia de Cabeça e Pescoço, Hospital Alemão Oswaldo Cruz, São Paulo, SP, Brasil; 4 Departamento de Cirugía de Cabeza y Cuello, Instituto de Oncología Ángel H. Roffo, Buenos Aires, Argentina; 5 Departamento de Cirugía, Hospital Pablo Tobón Uribe, Medellín, Colombia; 6 Centro de Tiroides, Rosario, Argentina; 7 Servicio de Cirugía de Cabeza y Cuello, Hospital Universitario Austral, Pilar, Argentina; 8 Departamento de Cirurgia de Cabeça e Pescoço e Otorrinolaringologia, A.C.Camargo Cancer Center, São Paulo, SP, Brasil; 9 Departamento de Cirurgia de Cabeça e Pescoço, Faculdade de Medicina, Universidade de São Paulo, São Paulo, SP, Brasil

**Keywords:** thyroidectomy, hemorrhage, laryngeal nerve injuries, hypoparathyroidism, health care technology

## Abstract

Thyroidectomyis the most frequent endocrine surgical treatment for problems such as goiter, thyroid cancer, and Graves’ disease. The global incidence of goiter ranges from 5%-20%, with a notably high frequency in less wealthy countries, and the incidence of thyroid cancer is on the rise due to the greater use of diagnostic imaging. Despite medical options, surgery remains essential. Surgical advancements such as blood vessel sealing technology, intraoperative laryngeal nerve neuromonitoring (IONM), remote access surgery, and parathyroid fluorescence have transformed thyroid surgery. Vessel sealing technologies reduce operative time and blood loss, whereas IONM preserves the laryngeal nerves. Remote access surgery, which includes a variety of techniques, produces results similar to those of open thyroidectomy with a longer operative time. Fluorescence enhances parathyroid detection and lowers the risk of temporary hypoparathyroidism. Economic studies reveal cost discrepancies, with advantages particularly visible in health care systems that depend on surgical time. While these advancements promise better patient outcomes, their accessibility and cost-effectiveness remain issues, particularly in Latin America. Recognizing these concerns, the Latin American Thyroid Society’s Surgical Affairs Committee conducted an extensive review of emerging thyroid surgery technologies to guarantee their proper use in the area.

## INTRODUCTION

Thyroidectomy is the most common procedure in endocrine surgery. The incidence of goiter ranges from 5%-20% globally, fluctuates according to the degree of iodine deficiency, and is high in developing nations. In a significant proportion of symptomatic patients or those with massive goiters, surgery provides rapid and effective treatment (^1^). Thyroid cancer is the most widespread endocrine cancer and one of the most common malignancies. Its prevalence continues to rise, primarily due to the indiscriminate utilization of diagnostic images. Differentiated thyroid cancer (DTC) is the most common malignancy, and patients with DTC are considered to have favorable prognosis and a high overall survival rate. Surgery remains the mainstay treatment for thyroid cancer (^2^). Graves’ disease can ultimately be treated with antithyroid drugs, radioactive iodine, and surgery. Nevertheless, surgery has the highest success rates for both primary and persistent diseases (^3^).

Traditionally, the thyroid gland is removed via a small incision made approximately 4-5 cm above the sternal notch. Hypoparathyroidism, recurrent laryngeal nerve (RLN) injury, and neck hematoma are the most common complications. New technologies have had a positive impact on the ability to diagnose and treat a variety of surgical conditions, and in thyroid surgery, they appear to offer benefits in terms of reduced risk, increased accuracy, and enhanced quality of life. To minimize operative risks, these technologies utilize advanced imaging modalities and minimally invasive surgical procedures. Before they can be widely implemented, these new devices are subject to regulatory approval to assure patient safety. However, despite regulatory controls, the use of these technologies may introduce unexpected risks into treatment and have ethical implications on the accessibility of services and the cost of treatment (^4^).

More than two decades have passed since the introduction of devices that improve hemostasis, reduce the risk of laryngeal nerve and parathyroid injuries, and make it possible to offer invisible scar surgeries in thyroid surgery (^5^). Several of these innovations have been disseminated and implemented in developed countries with clinical, social, cultural, and economic conditions that are distinct from those of Latin America. Supported by the concept of value-based care, the Surgical Affairs Committee of the Latin American Thyroid Society (LATS) developed this expert opinion document to evaluate the efficacy and feasibility of implementing novel technologies in thyroid surgery (^6^).

This expert opinion document has been divided into two parts for this purpose. The first section focuses on the most commonly used techniques in thyroid surgery: vessel sealing, neuromonitoring, parathyroid fluorescence, and remote access surgery. In the second section, the use of thermal ablation as a primary treatment strategy for benign and malignant thyroid nodules is discussed.

These recommendations, which are based on the best evidence available, seek reasonable and targeted usage in circumstances where the most benefits may be found with the highest safety and lowest cost, while taking into consideration the unique characteristics of health services.

These expert opinion recommendations may differ from those published by other surgical societies since they aim to provide a regional viewpoint based on distinctive features. However, the suggestions made in this study give Latin American surgeons flexibility of choice.

## VESSEL SEALING TECHNOLOGY

### Description of the technology

Vessel sealing technology involves devices that coagulate blood vessels without the need for sutures or clips. There are two popular technologies: one uses ultrasonic energy and seals vessels by a mechanical effect on the walls (Harmonic^®^), and the other uses thermal energy and seals by electrocoagulation, also known as an advanced bipolar sealer (LigaSure^TM^).

### Bibliographic research

To date, twelve systematic reviews have been published, and nine were included in this evaluation (^7-15^). These studies are related to the evaluation of vessel sealing systems in thyroid surgery, the first of which was in 2010. The excluded studies and their reasons for being excluded are included in the supplementary material.

### Effectiveness

In general, vessel sealing devices offer advantages over the traditional approach of cutting and ligation due to a 25-minute reduction in surgical time, which varies depending on whether a total thyroidectomy or lobectomy is being performed. In addition, they reduce intraoperative blood loss by 15-25 mL and postoperative drainage by 15-25 mL. Adjusting for confounding variables, Garas and cols. (^11^) reported that decreases in operative time and bleeding volume were statistically significant. Contin and cols. (^9^) assessed surgical time in relation to the type of thyroidectomy. They reported a decrease of 24.9 minutes in total thyroidectomy and 17.4 minutes in lobectomy when an ultrasonic device was utilized as opposed to ligation. For patients receiving treatment using an advanced bipolar sealer, the reduction in total thyroidectomy time was 16.4 minutes, and the reduction in lobectomy time was 15 minutes. Similar assessments were made for reoperation, operative site infection, pain and cosmetic satisfaction. Luo and cols. (^13^) reported that an ultrasonic scalpel reduced the total thyroidectomy time by 26.3 minutes and lobectomy time by 21.9 minutes, whereas using an advanced bipolar sealer reduced these times by 12.7 and 17.7 min, respectively **(Table 1)**.

**Table 1:** Characteristics of the systematic reviews evaluating sealing vessels devices

Study	Time	Number of included studies	Type of included studies	Total number of patients	Comparison Harmonic vs. Conventional						
					Operative time	Bleeding	Drainage	Seroma	Complications	Pain	Length of stay
Ecker and cols.	1966-2008	12	RCT	1,153	Decrease 23.4 min^*^	Decrease 24.2 mL^*^	Decrease 9.2 mL		ND	Decrease 0.86 score VAS^*^	Decrease 0.12 days^*^
Garas and cols.	2000-2012	35	RCT	2,856	H vs. C Decrease 1.29 SMD^*^	H vs. C Decrease 1.22 SMD^*^	H vs. C Decrease 0.36 SMD^*^	H vs. C ND	Hip H vs. C ND		H vs. C ND
					L vs. C Decrease 1.08 SMD^*^	L vs. C ND	L vs. C ND	L vs. C ND	Hip L vs. C ND		L vs. C ND
									RLNI H vs. C ND		
									RLNI L vs. C ND		
Contin and cols.	2000-2012	35	RCT	4,061	H vs. C Decrease 22.2 min^*^	H vs. c Decrease 28.5 mL^*^	H vs. C Decrease 11.2 mL^*^	ND	RLNI ND		H vs. C Decrease 0.28 days^*^
					L vs. C Decrease 13.8 min^*^	H vs. L ND	L vs. C ND		Hip ND		L vs. C ND
Cheng and cols.	2000-2014	14	RCT	2,516	H vs. C Decrease 29.13 min^*^	H vs. C 45.4 mL^*^	H vs. C Decrease 29 mL^*^	H vs. C ND	H vs. C Bleeding ND	H vs. C Decrease 1.33 score VAS	H vs. C Decrease 0.68 days^*^
									H vs. C Hip ND		
									H vs. C RLNI ND		
Revelli and cols.	2003-2014	21	RCT	3,135	H vs. C Decrease 25.4 min^*^	H vs. C Decrease 30.5 mL^*^	H vs. C Decrease 12.9 mL^*^	H vs. C ND	H vs. C Hip ND	H vs. C Decrease 0.87 score VAS	ND
									H vs. C RLNI ND		
					H vs. C Decrease 25.9 min^*^		H vs. C ND		H vs. C Decrease hip temporal OR 0.56 (0.39-0.81)^*^	H vs. C Decrease 1.33 score VAS^*^	H vs. C Decrease 0.52 days^*^
									H vs. C Decrease RLNI ND		
Cannizzaro and cols.	2008-2014	14	RCT	2,293	Decrease 27.2 min^*^	Decrease 17.8 mL^*^			ND	Decrease 1.88 score VAS^*^	Decrease 0.5 days^*^
Luo and cols.	1966-2015	47	RCT-NRCT	6,219	H vs. C Decrease 24.2 min^*^	H vs. C Decrease 36.1 mL^*^	ND	ND	H vs. C Decrease RLNI OR 0.27 (0.1-0.7)^*^		
					L vs. C Decrease 13.1 min^*^	L vs. C Decrease 14.7 mL^*^			L vs. C ND		
Zhang and cols.	1966-2016	7	RCT-NRCT	813	L vs. C Decrease 17.4 min^*^	L vs. C Decrease 14.3 mL^*^	L vs. C Decrease 6.4 mL^*^		RLNI ND		
									Hip temp Decrease OR 0.49 (0.27-0.9)^*^		
Hua and cols.	1966-2018	23	RCT	5,408		ND					
Ecker and cols.	1966-2008	12	RCT	1,153	Decrease 35 min^*^	Decrease 14 mL^*^					
Garas and cols.	2000-2012	35	RCT	2,856	ND	ND	ND	ND	Hip ND		ND
									RLNI ND		
Contin and cols.	2000-2012	35	RCT	4,061	Decrease 8.42 min^*^	ND	ND	ND	RLNI ND		ND
									Hip ND		
Cheng and cols.	2000-2014	14	RCT	2,516							
Revelli and cols.	2003-2014	21	RCT	3,135							
Cannizzaro and cols.	2008-2014	14	RCT	2,293	ND	ND			ND	Decrease 0.4 score VAS^*^	ND
Luo and cols.	1966-2015	47	RCT-NRCT	6,219	ND	ND	ND	ND	ND		
Zhang and cols.	1966-2016	7	RCT-NRCT	813	ND	ND	ND		ND		
Hua and cols.	1966-2018	23	RCT	5,408		ND					

* Statistically significant.

RCT: randomized controlled trial; NRCT: not-randomized controlled trial; H: harmonic scalpel; C: conventional; VAS: visual scale analogue; ND: no difference; SMD: standardized median difference; Hip: hypoparathyroidism; RLNI: recurrent laryngeal nerve injury; Temp: temporary.

### Safety

The assessment of complications, such as definitive hypoparathyroidism and definitive RLN injury, was comparable between the groups. Some studies indicate that these devices reduce the risk of temporary RLN injury or transient hypocalcemia, but the results are inconsistent. Therefore, these devices are safe and have the same complication rate as the conventional method.

### Accessibility and costs

Garas and cols. (^11^) reported a statistically significant reduction in favor of the ultrasonic scalpel compared with the standard ligation technique, with a value of 0.57 times the standardized mean, but no differences were observed in comparison with the advanced bipolar technique.

The search revealed four studies that evaluated the costs of utilizing devices (^16-19^). Ortega and cols. (^16^) evaluated the use of the device in an endocrine surgery center in Spain and, in a study of total costs, reported that the use of the device significantly decreased the value of the procedure (vessel sealing €988 *vs.* traditional technique €1148). In a study conducted in France, Sebag and cols. (^17^) determined that the use of the devices did not result in any differences in total costs (vessel sealing €1024 *vs.* traditional technique €990). In Pakistan, Bhettani and cols. (^18^) reported that the use of the device increased the cost of the procedure (vessel sealing 190 PKR *vs.* traditional technique 138 PKR). Despite between-group adjustments, Konishi and cols. (^19^), in a study in Japan, reported a statistically significant difference between the cost of using the device and the cost of using the traditional technique (vessel sealing US$ 7,412 versus US$ 6,765). In Latin America, no specific studies on the costs of using the device were found. Kowalski and cols. (^20^), in a clinical trial evaluating the use of an ultrasonic scalpel in Brazil, reported a 14% difference in favor of the device, but this difference was not statistically significant.

### Comments

Systematic reviews do not discriminate by type of pathology, and in some instances, more complex procedures, such as neck dissection, are included, so most results are heterogeneous and should be analyzed with caution.

Most studies indicate that total thyroidectomy takes between 45 and 150 minutes and that partial thyroidectomy takes between 75 and 110 minutes. This means that the reduction in surgical time is between 20% and 30% of the average time for an open procedure, depending on the complexity of the procedure and the surgeon’s experience. For health care systems where procedure costs are dependent on surgical time, the benefit appears more evident. In systems where the value of the procedure is independent of the surgical time (payment by activity), however, the benefit must be evaluated against the cost of the device. In terms of the volume of intraoperative bleeding, the reported averages for the conventional technique are close to 80 mL, so a reduction of 15 to 25 mL could be considered insignificant. A similar analysis could be performed on postoperative drainage, especially since there is evidence that the routine use of drains (^21^) and hemostatics (^22^) after thyroid surgery does not offer any benefits in terms of postoperative collection or bleeding. Finally, while data are limited, surgeons who use fewer devices, such as forceps and clamps, may benefit from improved ergonomics.

In Latin America, the value of devices differs across countries. In addition, these devices have varying reuse policies. In accordance with the manufacturer’s instructions, these devices are intended for a single use; however, depending on the country and health institution, they are used multiple times.

If the use of a vessel sealing device eliminates the need for routine drainage or hemostatic agents, the patient (comfort, length of hospital stay) and the system may experience benefits (similar or lower costs). As most health care systems in Latin America have surgical time constraints and are overloaded, a reduction in the operative time associated with the device could reduce waiting times for a surgical procedure. Its routine use is recommended for situations where a prolonged operative time is anticipated or where there is a risk of bleeding and major seroma (for example, giant goiters, hyperthyroidism, locally invasive cancer, total thyroidectomy with lateral neck dissection, remote access thyroidectomy (RAT), etc.). In the case of other conditions, surgeons are free to use it if it is available and if they understand it may benefit each specific case.

## NEUROMONITORING OF THE LARYNGEAL NERVE

### Description of the technology

Intraoperative laryngeal nerve neuromonitoring (IONM) is based on the electrophysiological principle of stimulation and measurement of the target organ’s response. In this case, there is a stimulating electrode that administers energy to the nerve and a receptor electrode that measures the response of the intrinsic muscles of the larynx to nerve stimulation. Currently, there are two methods of neuromonitoring: intermittent, in which the stimulus is applied to the nerve only when necessary, and continuous, in which the stimulating electrode is implanted in the vagus nerve and generates pulses periodically and at a predetermined frequency.

### Bibliographic research

Fourteen systematic reviews (^23-36^) were included. The excluded studies and their reasons for exclusion are included in the supplementary material.

### Effectiveness

Except for the meta-analysis by Bai and Chen (^33^), the routine use of IONM does not reduce the incidence of definitive RLN injury in thyroidectomy patients. Five meta-analyses demonstrated a decrease in transitory RLN injury, whereas the others failed to demonstrate an effect (^27,31-33,37^). Only one study (^24^) reported a reduction in transient superior laryngeal nerve injury, and there was no evidence of an effect on permanent superior laryngeal nerve injury. The two studies evaluating surgical time (^26,34^) did not reveal statistically significant differences.

Pisanu and cols. (^26^) evaluated the subgroups of high-risk and low-risk patients and reported no statistically significant differences between them. Lombardi and cols. (^28^) evaluated the results according to the methodological design (RCTs versus nonrandomized comparative studies) and the follow-up duration to define vocal paralysis (6 months versus 12 months) but did not find statistically significant differences. Pardal-Refoyo and Ochoa-Sangrador (^29^) assessed only the occurrence of bilateral paralysis and discovered a reduced frequency in the IONM group. Sun and cols. (^30^) evaluated only patients who underwent reoperation and discovered a reduction in the definitive RLN lesion. Wong and cols. (^31^) included only high-risk patients and discovered differences in temporary but not permanent RLN injury. Ku and cols. (^35^) evaluated continuous neuromonitoring and reported a lower rate of laryngeal nerve paralysis **(Table 2)**.

**Table 2:** Characteristics of the systematic reviews evaluating intraoperative neuromonitoring

Study	Time	Number of included studies	Type of included studies	Number or laryngeal nerves at risk	Definitive injury of RLN	Temporary injury of RLN	Definitive injury of SLN	Temporary injury of SLN	Operative time
Higgins and cols.	1966-2008	42	RCT-NRCT-Case series	64,817	ND	ND			
Sanabria and cols.	1966-2012	6	RCT	3,064	ND	ND	ND	Decrease (RD -4% (-8 a -1%)	
Zheng and cols.	1966-2011	14	RCT-NRCT	3,6487	ND	Decrease (OR 0.8) (0.65-0.99)			
Pisanu and cols.	2004-2013	20	RCT-NRCT	35,513	ND	ND			ND
Rulli and cols.	1994-2012	8	RCT-NRCT	5,257	ND	Decrease (RR 0.73) (0.54-0.98)			
Lombardi and cols.	1966-2014	14	RCT-NRCT	38,820	ND	ND			
Wong and cols.	2000-2015	10	RCT-NRCT	10,615	ND	Decrease (OR 1.47) (1.07-2.0)			
Yang and cols.	2004-2016	24	RCT-NRCT	17,203	ND	Decrease (OR 0.76) (0.61-0.94)			
Bai and Chen	1980-2017	34	RCT-NRCT	60,247	Decrease (RD -0.26% (-0.39 a -0.12%)	Decrease (RR 0.71) (0.57-0.88)			
Cirocchi and cols.	1966-2018	5	RCT	2,895	ND	ND			ND
Davey and cols.	1966-2021	8	RCT	4,977	ND	ND			

RCT: randomized controlled trial; NRCT: not-randomized controlled trial; ND: no difference; RLN: recurrent laryngeal nerve; SLN: superior laryngeal nerve.

### Safety

Only the review by Cirocchi and cols. (^34^) addressed the measurement of adverse events and concluded that there was no increase in adverse events. There are reports of hemodynamic effects resulting from continuous vagus nerve stimulation (^38^), but others have not reported an increase in these effects (^39-41^). In cases of electrophysiological or hemodynamic abnormalities, cessation of the procedure permitted complete recovery, with no fatalities or long-term effects reported.

### Accessibility and costs

We discovered seven studies that evaluated the cost-effectiveness of IONM. Using secondary data from the United States, Al-Qurayshi and cols. (^42^) reported that IONM was cost-effective for total thyroidectomy at twenty years, whereas Wang and cols. (^43^) did not find IONM to be cost-effective in a similar study conducted in Italy. Three studies of cost analysis in Italy (^44-46^) reported that IONM increased the cost of the procedure by 5%-9%, with benefits in terms of a reduction in indirect costs. Conversely, Sanguinetti and cols. (^47^) discovered that the use of IONM increased costs within the Italian health care system. Gremillion and cols. (^48^) compared costs in two groups of patients in the United States and reported that IONM increased costs with no impact on effectiveness. There was only one economic evaluation conducted in Latin America. Sanabria and Ramirez (^49^) were unable to demonstrate the cost-effectiveness of routine IONM in Colombia.

### Comments

Most of the evaluated systematic reviews included patients who were candidates for total thyroidectomy but who had diverse pathologies (goiter, hyperthyroidism, malignancy) with different risks of laryngeal nerve injury. In general, children, patients who underwent central neck dissection or intrathoracic goiter or who underwent reoperations, which have a relatively high risk of RLN injury, were not included; consequently and unfortunately, the findings cannot be extrapolated to these patients. Most studies have focused on the evaluation of RLN injury, whereas only a few have investigated the more common superior laryngeal nerve injury.

In general, the evaluated reviews did not demonstrate a statistically significant reduction in definitive lesions of the RLN, but some demonstrated a statistically significant reduction in transitory injuries. Only one systematic review assessed the effect on the superior laryngeal nerve and reported similar results. The evaluation of the impact on surgical time did not reveal statistically significant differences.

The available data allow us to assert that the device is safe and that hemodynamic alterations may eventually occur, particularly in the case of continuous IONM, which are reversible and have not been associated with an increase in morbidity or mortality.

Economic studies are heterogeneous. In the United States, studies have demonstrated that IONM is cost effective in this health system but not in Italy. In the absence of a cost-effectiveness evaluation, direct cost studies demonstrated a cost increase for IONM-based procedures, which would be mitigated by a reduction in indirect costs over time. Only one economic evaluation was found for Latin American countries, and due to the obvious differences in health systems between nations, these conclusions cannot be extrapolated directly and must be evaluated with caution. The value of the device also varies across countries in Latin America.

The use of IONM could decrease the frequency of transient lesions of the laryngeal nerves, which is essential for deciding whether to perform a staged thyroidectomy in the event of a loss of nerve signal without intraoperative signal recovery, thereby eliminating the possibility of bilateral nerve injury and subsequent tracheotomy. However, the results of these studies are inconclusive in favor of or against their effects. Recent research has indicated that the ability of continuous neuromonitoring to anticipate laryngeal nerve traction injuries could reduce the incidence of laryngeal injuries. However, comparative investigations between intermittent IONM and conventional surgery are scarce. IONM revealed no differences in operative time. As its effect on definitive RLN injury is unknown, routine neuromonitoring should be carefully evaluated. Its use is recommended in situations where there is an increased risk of laryngeal nerve injury (for example, giant or endothoracic goiters, hyperthyroidism, locally advanced malignancy, reoperation, etc.). Similarly, it should be used for patients who need voice communication to work, especially voice professionals such as singers, teachers, journalists, and call center operators, for whom a transient recurrent paralysis would be negative. Regardless, there is a lack of evidence of a protective effect in cases of reoperation; since central compartment reoperations carry a greater risk of RLN injury, IONM should be considered wherever available.

## REMOTE-ACCESS THYROIDECTOMY

### Description of the technology

All surgical procedures that are facilitated by endoscopy through access routes other than the traditional cervical thyroidectomy incision are categorized as remote-access thyroidectomy (RAT). The transaxillary (TAA, through endoscopic ports located in the axilla), transmammary (BABA, through endoscopic ports located around the nipples), retroauricular (RA, through an incision in the retroarticular hair line and assisted by endoscopic instruments), and transoral (TOETVA, through endoscopic ports located in the oral vestibule) accesses are included. The robot can assist with any of the processes, and gas may or may not be used to dissect the tissues and keep the surgical field open. Among the most widely performed remote access procedures, TOETVA is the most popular.

### Bibliographic research

To date, 28 systematic reviews have been published (^25,50-65^), and 17 were included in this evaluation. The excluded studies and their reasons for being excluded are included in the supplementary material.

### Efficiency and safety

Most of the studies were retrospective nonrandomized comparative studies conducted in Asia. Both total thyroidectomy and lobectomy have been the subject of research, and a significant number of studies also included concomitant central neck dissection. A considerable number of articles compared robotic and open surgery (^25,55-59,62,65,66^).

All meta-analyses confirmed that RAT resulted in the same number of definitive RLN lesions as the open technique did, and some reported an increase in transient RLN injuries (two studies involving endoscopic TAA-BABA and one study involving robotic TAA-BABA) (^53,59,63^). With respect to definitive and transient hypoparathyroidism, all studies confirmed a comparable number between the techniques, with the exceptions of Liu and cols. (^59^) and Jiang and cols. (^63^), who reported a lower number in patients receiving RAT. All investigations demonstrated a comparable incidence of postoperative hemorrhage.

With the exception of Son and cols. (^57^), the greatest difference between the techniques is the increase in surgical time by 30%-50% compared with the open technique. In the studies by Chen and cols. (53 and Jiang and cols. (^63^), the duration of stay was reported to be longer. A small number of studies on tumor recurrence reported no difference between treatment methods (^53,58-60,63,66^) **(Table 3)**.

**Table 3:** Characteristics of the systematic reviews evaluating remote access surgery

Study	Time	Number of included studies	Type of included studies	Total number of patients	Surgical technique	Remote access	Open surgery	TT	TT/TP	M/B	M	Neck dissection
Li and cols.	1968-2013	6	CNA	1,101	Endoscopic^*^	613	468	NR	NR	0	1,101	137
Chen and cols.	1968-2019	12	CNA	2,672	Endoscopic TAA	799	2,672	1,682	990	0	2,672	1,892
Jiang and cols.	1968-2019	20	RCT, CNA	5,664	Endoscopic TAA-BABA	1,633	4,031	5,664	0	0	5,664	3,842
Jasaitis and cols.	1968-2020	10	RCT, CNA	1,597	Endoscopic TAA	534	1,057	1,375	216	536	1,061	NR
de Vries and cols.	NR	31	RCT, CNA	25,373	Endoscopic BABA-TAA-RA-TOETVA	1,927	2,891	1,115	15,319	9,592	15,781	12,280
Jackson and cols.	1968-2011	9	RCT, CNA	2,881	Robotic/Endoscopic BABA-TAA	2,087	794	NR	NR	NR	NR	NR
Sun and cols.	1968-2013	11	CNA	1,931	Robotic BABA-TAA	726	1,205	1,327	604	160	1,771	NR
Shen and cols.	1990-2013	9	CNA	1,615	Robotic TAA	510	1,105	NR	NR	NR	NR	NR
Lang and cols.	1968-2014	10	CNA	2,205	Robotic BABA-TAA	752	1,453	1,757	448	0	2,205	NR
Son and cols.	1968-2014	14	CNA	3,136	Robotic BABA-TAA	1,066	2,070	1,813	1,323	0	3,136	2,977
Pan and cols.	1968-2016	23	CNA	5,200	Robotic BABA-TAA	1,933	3,267	4,274	926	0	5,200	5,025
Xing and cols.	1968-2019	30	CNA	6,622	Robotic BABA-TAA	2,401	4,221	NR	NR	63	6,559	4,503
Liu and cols.	1968-2020	59	RCT, CNA	11,947	Robotic BABA-TAA	4,593	7,420	8,382	3,565	0	11,947	NR
Kang and cols.	1968-2022	10	CNA	1,420	TOETVA	NR	NR	NR	NR	1,420	0	NR

* Endoscopic technique not described.

TAA: transaxillary; BABA: transmammary; RA: retroauricular; TOETVA: transoral.

**Table:** 

Study	Definitive injury of RLN	Temporary injury of RLN	Definitive hypoparathyroidism	Temporary hypoparathyroidism	Hematoma	Operative time	Length of stay	Recurrence
Li and cols.	ND	Higher in ET^*^	ND	ND	NR	Higher in ET^*^	ND	ND
Chen and cols.	ND	Higher in ET^*^	ND	ND	ND	Higher in ET^*^	Higher in ET^*^	ND
Jiang and cols.	ND	Higher in ET^*^	ND	Lower in ET^*^	NR	Higher in ET^*^	ND	ND
Jasaitis and cols.	ND	ND	ND	ND	ND	Higher in ET^*^	Lower in ET^*^	NR
de Vries and cols.	ND	ND	ND	ND	NR	Higher in ET^*^	ND	NR
Jackson and cols.	NR	NR	NR	NR	NR	Higher in RT^*^	ND	NR
Sun and cols.	ND	ND	ND	ND	ND	Higher in ET^*^	ND	NR
Shen and cols.	ND	ND	ND	ND	ND	Higher in ET^*^	ND	NR
Lang and cols.	NR	NR	NR	NR	NR	NR	NR	ND
Son and cols.	ND	ND	ND	ND	ND	ND	NR	NR
Pan and cols.	ND	ND	ND	ND	NR	Higher in ET^*^	ND	ND
Xing and cols.	NR	NR	ND	ND	NR	Higher in ET^*^	ND	NR
Liu and cols.	ND	ND	Lower in ET^*^	ND	ND	Higher in ET^*^	ND	ND
Kang and cols.	ND	ND	ND	ND	ND	Higher in ET^*^	NR	NR

* Statistically significant.

RCT: randomized controlled trial; NRCT: not-randomized controlled trial; ND: no difference; RLNI: recurrent laryngeal nerve injury; TT: total thyroidectomy; PT: partial thyroidectomy; M: malignant; B: benign; ET: endoscopic technique; RT: robotic technique; NR: not reported.

### Accessibility and costs

None of the systematic reviews evaluated cost-effectiveness information. There were no economic cost-effectiveness studies found, and the vast majority were merely cost comparisons.

In 2012, Cabot and cols. (^67^) compared the costs of open thyroidectomy (OT) and transaxillary endoscopy and reported that endoscopic thyroidectomy was more expensive ($3,000-$4,000). Razavi and cols. (^68^) analyzed the direct costs of transoral thyroidectomy and reported a difference of approximately $1,200, with RAT being more expensive. This study did not evaluate additional costs.

### Comments

In most of the systematic reviews evaluated, patients who were candidates for total or partial thyroidectomy via remote access were compared with patients who underwent total thyroidectomy in nonrandomized retrospective or prospective studies. The studies were much clearer in defining precise inclusion criteria for remote access surgery (small tumors, without suspicion of high-risk conditions, in patients with favorable clinical and anatomical conditions) but not for total thyroidectomy, which is currently indicated for all patient types. This reveals selection bias in the majority of the studies, which should lead to uncertain conclusions.

In general, the evaluated reviews did not reveal a statistically significant difference between the risks of temporary or permanent RLN injury, temporary or permanent hypoparathyroidism, or hematoma. Those who evaluated oncological outcomes also failed to demonstrate the difference between OT and its alternatives. The only consistent difference was an increase in surgical time. However, information suggests that surgical time decreases over time and after the learning curve has been reached. Thus, the available data allow us to assert that the RAT is safe and yields comparable outcomes to OT in patients who satisfy the inclusion criteria outlined previously. However, they require surgeons with specific training and who have mastered the learning curve to perform them. This conclusion cannot be generalized to all thyroid surgery patients.

There are few economic analyses available. The few studies that have already been performed in the United States demonstrated that, compared with OT, remote access techniques increase total costs. However, these studies did not compare clinical outcomes or their relationship with cost. These conclusions cannot be directly extrapolated and must be evaluated with caution due to the evident differences in health systems between countries.

In addition to robotic surgery, the devices required to perform remote access surgery may not be available at many institutions in Latin America. In other cases, remote access surgery requires endoscopic devices (cameras, lenses, and trocars), such as those used in laparoscopic abdominal surgery, which are accessible, but also requires the use of endoscopic vessel-sealing devices (ultrasonic scalpels), although there are reports of the use of bipolar energy devices. In addition, the increasing length of surgical time also contributes to an increase in total cost, but it can decrease with time. Some authors advocate the mandatory use of neuromonitoring of the RLN, the use of special devices to generate a virtual workspace (balloons), and the routine use of hemostats, which are selectively used in open surgery. However, the use of special devices to generate a virtual workspace is not mandatory and can be replaced by other low-cost methods or the use of only hook cautery.

Since the greatest benefit of RAT is cosmetic, it must be weighed against the availability of resources and the specific requirements of health systems and can be considered an alternative to standard management practices in Latin America.

## FLUORESCENCE FOR PARATHYROID PRESERVATION

Two fluorescence methods have been evaluated clinically. The first method employs intravenous infusion of indocyanine green (ICG) and subsequent identification with a special lamp, and the second method uses autofluorescence, which takes advantage of the ability of parathyroid tissue to emit fluorescence at a specific range of the light spectrum, which is emitted by a source designed specifically for this purpose. A camera or a probe can be used to detect fluorescence. The objective is to identify parathyroid gland vascularization and to indicate intraoperative reimplantation in those with a lack of blood flow.

### Bibliographic research

To date, 7 systematic reviews have been published (^69-72^) related to the evaluation of fluorescence as an adjuvant in the identification of parathyroid glands in thyroid surgery. The excluded studies and their reasons for being excluded are included in the supplementary material.

### Effectiveness and safety

Most of the studies reported in the literature are retrospective nonrandomized comparative studies conducted in Asia.

One meta-analysis (^72^) revealed that fluorescence enhances the intraoperative identification of parathyroid glands compared with open surgery, whereas another study reported no significant difference (^71^). Fluorescence consistently reduces the incidence of transient hypoparathyroidism, but no statistically significant differences in the incidence of definitive hypoparathyroidism have been identified **(Table 4)**.

**Table 4:** Characteristics of the systematic reviews evaluating fluorescence to identify parathyroid glands

Study	Time	Number of included studies	Type of included studies	Total number of patients	Fluorescence technique	Fluorescence	Control	Intraoperative identification	Temporary hypoparathyroidism	Definitive hypoparathyroidism
Barbieri and cols.	1968-2020	13	RCT, NRCT	1,484	VI/AF	597	887	NR	Higher in control^*^	ND
Weng and cols.	2011-2021	6	RCT, NRCT	2,180	AF	493	1,687	NR	Higher in control^*^	ND
Wang and cols.	1968-2021	7	RCT, NRCT	1,480	AF	NR	NR	ND	Higher in control^*^	ND
Lu and cols.	1968-2021	8	RCT, NRCT	2,889	AF	844	2,045	Higher in fluorescence^*^	Higher in control^*^	ND

* Statistically significant.

RCT: randomized controlled trial; NRCT: not-randomized controlled trial; ND: no difference; NR: not reported; VI: visual identification.

There are no reports indicating that the use of fluorescence increases adverse events.

### Accessibility and costs

None of the systematic reviews evaluated cost-effectiveness information. No cost-effectiveness studies were found.

### Comments

Retrospective comparative studies and some randomized clinical trials were included in the systematic reviews that were analyzed. Although gland identification has effects on transitory hypoparathyroidism, no advantages have yet been identified in definitive hypoparathyroidism, as indicated by most studies.

There is no information on the costs associated with the use of technology or whether the necessary equipment is available in Latin America.

Before recommending its routine use, additional information is needed.

## Supplementary material

**Table 1:** Excluded systematic review or meta-analysis and reasons

Vessel sealing technology	
Zhao JZ, Gao M, Yu Y, et al. [Harmonic scalpel versus conventional resection in thyroid surgery: a meta analysis on the safety outcomes]. Zhonghua Er Bi Yan Hou Tou Jing Wai Ke Za Zhi. 2013;48(^9^):752-757.	Chinese language
Pacilli M, Tartaglia N, Gerundo A, Pavone G, Fersini A, Ambrosi A. Energy Based Vessel Sealing Devices in Thyroid Surgery: A Systematic Review to Clarify the Relationship with Recurrent Laryngeal Nerve Injuries. Medicina (Kaunas). 2020;56(^12^). doi:10.3390/medicina56120651	Narrative review
Spartalis E, Giannakodimos A, Ziogou A, et al. Effect of energy-based devices on post-operative parathyroid function and blood calcium levels after total thyroidectomy. Expert Rev Med Devices. 2021;18(^3^):291-298. doi:10.1080/17434440.2021.1899805	Narrative review
Neuromonitoring of laryngeal nerve	
Li K, Li J. [Meta analysis of the real-time nerve monitoring in prevention of recurrent laryngeal nerve injury during thyroid surgery]. Lin Chuang Er Bi Yan Hou Tou Jing Wai Ke Za Zhi. 2014;28(^24^):1941-1944, 1948.	Chinese language
Malik R, Linos D. Intraoperative Neuromonitoring in Thyroid Surgery: A Systematic Review. World J Surg. 2016;40(^8^):2051-2058. doi:10.1007/s00268-016-3594-y	Narrative review
Dionigi G, Donatini G, Boni L, et al. Continuous monitoring of the recurrent laryngeal nerve in thyroid surgery: a critical appraisal. Int J Surg. 2013;11 Suppl 1:S44-46. doi:10.1016/S1743-9191(^13^)60014-X	Narrative review
Naytah M, Ibrahim I, da Silva S. Importance of incorporating intraoperative neuromonitoring of the external branch of the superior laryngeal nerve in thyroidectomy: A review and meta-analysis study. Head Neck. 2019;41(^6^):2034-2041. doi:10.1002/hed.25669	Other population
Kim DH, Kim SW, Hwang SH. Intraoperative Neural Monitoring for Early Vocal Cord Function Assessment After Thyroid Surgery: A Systematic Review and Meta-Analysis. World J Surg. 2021;45(^11^):3320-3327. doi:10.1007/s00268-021-06225-x	Other design
Remote access surgery	
Dabsha A, Khairallah S, Elkharbotly I, et al. Learning curve and volume outcome relationship of endoscopic trans-oral versus trans-axillary thyroidectomy; A systematic review and meta-analysis. Int J Surg. 2022;104:106739. doi:10.1016/j.ijsu.2022.106739	Only endoscopic techniques
Lang BH, Wong CK, Tsang JS, Wong KP. A systematic review and meta-analysis comparing outcomes between robotic-assisted thyroidectomy and non-robotic endoscopic thyroidectomy. J Surg Res. 2014;191(^2^):389-398. doi:10.1016/j.jss.2014.04.023	Only endoscopic techniques
Wang D, Wang Y, Zhou S, et al. Transoral thyroidectomy vestibular approach versus non-transoral endoscopic thyroidectomy: a comprehensive systematic review and meta-analysis. Surg Endosc. 2022;36(^3^):1739-1749. doi:10.1007/s00464-021-08836-w	Only endoscopic techniques
Akritidou E, Douridas G, Spartalis E, Tsourouflis G, Dimitroulis D, Nikiteas NI. Complications of Trans-oral Endoscopic Thyroidectomy Vestibular Approach: A Systematic Review. In Vivo. 2022;36(^1^):1-12. doi:10.21873/invivo.12671	Only endoscopic techniques
Chen S, Zhao M, Qiu J. Transoral vestibule approach for thyroid disease: a systematic review. Eur Arch Otorhinolaryngol. 2019;276(^2^):297-304. doi:10.1007/s00405-018-5206-y	Only endoscopic techniques
Martino B, Nitro L, De Pasquale L, et al. Conversion rates in robotic thyroid surgery: A systematic review and meta-analysis. Int J Med Robot. 2022;18(^5^):e2427. doi:10.1002/rcs.242	Only endoscopic techniques
Tartaglia F, Maturo A, Di Matteo FM, et al. Transoral video assisted thyroidectomy: a systematic review. G Chir. 2018;39(^5^):276-283.	Only endoscopic techniques
Haidar Ismail N, Tavalla P, Uppal P, et al. The Advantages of Robotic Over Open Thyroidectomy in Thyroid Diseases: A Systematic Review. Cureus. 2022;14(^6^):e26320. doi:10.7759/cureus.26320	Narrative review
Scerrino G, Melfa G, Raspanti C, et al. Minimally Invasive Video-Assisted Thyroidectomy: Analysis of Complications from a Systematic Review. Surg Innov. 2019;26(^3^):381-387. doi:10.1177/1553350618823425	Other design
Nechay TV, Panin SI, Sazhin AV, et al. [Comparison of robot-assisted and conventional endoscopic surgeries in the Russian Federation. (A systematic review and meta-analysis)]. Khirurgiia (Mosk). 2022(^6^):88-101. doi:10.17116/hirurgia202206188	Russian language
Fluorescence for parathyroid preservation	
Kim DH, Lee S, Jung J, Kim S, Kim SW, Hwang SH. Near-infrared autofluorescence-based parathyroid glands identification in the thyroidectomy or parathyroidectomy: a systematic review and meta-analysis. Langenbecks Arch Surg. 2022;407(^2^):491-499. doi:10.1007/s00423-021-02269-8	Without control group
Wang B, Zhu CR, Liu H, Yao XM, Wu J. The Accuracy of Near Infrared Autofluorescence in Identifying Parathyroid Gland During Thyroid and Parathyroid Surgery: A Meta-Analysis. Front Endocrinol (Lausanne). 2021;12:701253. doi:10.3389/fendo.2021.701253	Without control group
Kim DH, Kim SH, Jung J, Kim SW, Hwang SH. Indocyanine green fluorescence for parathyroid gland identification and function prediction: Systematic review and meta-analysis. Head Neck. 2022;44(^3^):783-791. doi:10.1002/hed.26950	Without control group
